# Effects of a Chilean Hazelnut-Supplemented Diet on Oxidative Stress and Cytokine Expression in PBMCs of Hypercholesterolemic Adults: A Randomized Controlled Trial

**DOI:** 10.3390/nu18203400

**Published:** 2026-10-16

**Authors:** Carlos González-Contreras, Sofía Ramos-Pardo, Esteban A. Valdebenito, Karina Ramírez-Alarcón, Felipe A. Zuniga, Maria Magdalena Quetglas-Llabrés, Antoni Sureda, Miquel Martorell

**Affiliations:** 1 Centro de Vida Saludable, Universidad de Concepción, Concepción 4070386, Chile; carlosalexgonza@udec.cl (C.G.-C.); sramos2016@udec.cl (S.R.-P.); evaldebenit2019@udec.cl (E.A.V.); 2 Departamento de Ciencias Biológicas, Facultad de Ciencias de la Vida, Universidad Andrés Bello, Talcahuano 4260000, Chile; 3 Departamento de Nutrición y Dietética, Facultad de Farmacia, Universidad de Concepción, Concepción 4070386, Chile; karramir@udec.cl; 4 Grupo en Nutrición Comunitaria y Estrés Oxidativo, Universidad de las Islas Baleares, 07122 Palma, Spain; m.quetglas@uib.es; 5 Departamento de Bioquímica Clínica e Inmunología, Facultad de Farmacia, Universidad de Concepción, Concepción 4070386, Chile; fzuniga@udec.cl; 6 Instituto de Investigación Sanitaria de las Islas Baleares (IdISBa), 07120 Palma, Spain; 7 CIBER Fisiopatología de la Obesidad y Nutrición (CIBEROBN), Instituto de Salud Carlos III (ISCIII), 28029 Madrid, Spain

**Keywords:** hypercholesterolemia, oxidative stress, *Gevuina avellana*, peripheral blood mononuclear cells, antioxidant enzymes, dietary intervention

## Abstract

Background/Objectives: Hypercholesterolemia is characterized by alterations in lipid metabolism, oxidative stress, and inflammatory status. Nuts have been shown to mitigate these alterations; however, limited evidence is available regarding their effects in the Chilean population using a native nut species. This study aimed to assess the impact of a six-month Chilean-adapted Mediterranean diet supplemented with *Gevuina avellana* Molina, compared with a low-fat regimen, on oxidative stress and inflammatory markers in peripheral blood mononuclear cells (PBMCs) from adults with hypercholesterolemia. Methods: A randomized controlled trial was conducted with a 6-month nutritional intervention. Of 106 hypercholesterolemic adults randomized, 81 completed the study, allocated to two groups: one following a Chilean diet supplemented with Chilean hazelnuts (n = 47) and the other a low-fat diet (n = 34) (ClinicalTrials.gov: NCT07087704). Oxidative stress markers and inflammatory markers in PBMCs were assessed as secondary outcomes. In PBMCs, reactive oxygen species (ROS) production and the enzymatic activities of superoxide dismutase (SOD), catalase (CAT), and glutathione reductase (GRd) were analysed, along with oxidative damage markers (malondialdehyde, carbonyls, and nitrotyrosine) and gene expression (SOD, CAT, GRd, IL-10, and TNF-α). Results: Both dietary interventions were associated with similar improvements in PBMC redox status, characterized by reduced oxidative damage and enhanced antioxidant defence capacity. Malondialdehyde and carbonyl levels decreased in both groups following the dietary intervention. In addition, antioxidant enzyme activities increased, with SOD showing a concomitant increase in gene expression level, whereas CAT gene expression decreased despite enhanced enzymatic activity. No significant differences between groups were observed for the assessed oxidative stress, antioxidant, or inflammatory markers. Conclusions: A Chilean diet supplemented with Chilean hazelnuts and a low-fat diet produced similar improvements in PBMC redox status in hypercholesterolemic adults. These findings suggest that improving dietary fat quality and increasing antioxidant-rich food intake may contribute to favourable changes in cellular redox homeostasis, although the specific contribution of Chilean hazelnuts requires further investigation.

## 1. Introduction

Cardiovascular diseases (CVDs) are the leading cause of death in Chile and worldwide, with ischemic heart disease and stroke being the most prevalent [1]. These conditions are closely linked to atherosclerosis, where hypercholesterolemia, particularly elevated low-density lipoprotein cholesterol (LDL-C), represents a major modifiable cardiovascular risk factor [2]. According to the 2016–2017 Chilean National Health Survey, 27.7% of the adult population presented high total cholesterol (TC > 200 mg/dL), and 5.2% had elevated LDL-C levels (>160 mg/dL) [3]. Hypercholesterolemia arises from a combination of genetic predisposition and modifiable lifestyle factors, particularly dietary factors, physical inactivity, and excess body weight [4]. Sustained LDL-C elevation promotes atherosclerotic plaque progression, increasing the risk of myocardial infarction, stroke, and peripheral artery disease [5]. Although statins represent the main pharmacological option, their use may be limited by adverse effects and suboptimal long-term adherence, underscoring the importance of dietary strategies as a first-line intervention [6].

A key recommendation for patients with hypercholesterolemia is lifestyle modification, including healthy diets as an essential strategy for reducing cardiovascular risk factors [7]. A commonly recommended approach is a low-fat diet, which restricts fat intake to 20–30% of total daily calories, emphasizing the consumption of fruits, vegetables, whole grains, seafood, and low-fat dairy while moderating portion sizes and promoting balanced energy intake according to individual requirements [8,9]. An alternative dietary pattern is the Mediterranean diet (MedDiet), which includes fruits, vegetables, whole grains, nuts, olive oil, fish, and moderate wine consumption. This dietary pattern is recognized for its lipid-lowering, anti-inflammatory, antioxidant, and cardioprotective effects, largely attributed to its high content of unsaturated fatty acids and bioactive polyphenols [10,11,12].

Nuts, rich in monounsaturated fatty acids (MUFA), antioxidants, vitamins, and minerals, have been associated with a reduced cardiovascular risk [13,14]. Among them, the Chilean hazelnut (*Gevuina avellana* Molina) stands out as the southernmost representative of the *Proteaceae* family, possessing an exceptional nutritional profile. It is predominantly composed of MUFA rather than saturated fatty acids (SFA) and contains tocopherols, phytosterols, vitamin E, essential minerals such as selenium, phenolic antioxidants, dietary fibre, and bioactive phytochemicals [14,15]. These characteristics give the Chilean hazelnut a nutritional profile similar to that of other tree nuts, such as almonds, walnuts, and European hazelnuts, which have been associated with cardiovascular benefits in epidemiological and intervention studies [13].

Evidence suggests that hazelnut consumption has beneficial effects on lipid profiles, oxidative stress, and inflammation without causing adverse effects. Various dietary interventions in hypercholesterolemic individuals have demonstrated significant reductions in LDL-C, triglycerides (TG), TC, and inflammatory markers, along with increases in high-density lipoprotein cholesterol (HDL-C) and antioxidants such as vitamin E [14,16,17]. Additionally, increases in the activity of antioxidant enzymes such as catalase (CAT) and superoxide dismutase (SOD), as well as modulation of PPARγ, a key regulator of inflammatory responses, have been observed [18]. A meta-analysis confirmed improvements in cardiometabolic and antioxidant factors without negatively affecting body weight, concluding that hazelnut-rich diets may provide sustained cardiometabolic benefits [19]. However, despite the recognized cardiometabolic properties of nuts, evidence regarding the effects of Chilean hazelnut consumption on cellular oxidative stress and inflammatory responses in humans remains limited, particularly at the level of peripheral blood mononuclear cells (PBMCs). Previous findings from this randomized trial demonstrated that both the Chilean hazelnut-supplemented diet and the low-fat diet improved the lipid profile and plasma antioxidant status in hypercholesterolemic adults [20]. However, whether these systemic improvements are accompanied by changes in cellular redox homeostasis and inflammatory signalling in circulating immune cells remains unclear. The present study extends those findings by specifically evaluating oxidative stress and cytokine responses in PBMCs.

The aim of this study was to examine the effects of a six-month dietary intervention comparing a Chilean diet supplemented with Chilean hazelnuts with a low-fat diet on oxidative stress and inflammatory markers in PBMCs from adults with hypercholesterolemia. PBMCs are increasingly used in nutritional research as accessible biomarkers of metabolic and inflammatory responses, as they reflect changes in immune and redox-related pathways associated with cardiometabolic disorders [21,22].

## 2. Materials and Methods

### 2.1. Subjects and Study Design

This study is a randomized controlled trial involving a six-month nutritional intervention carried out in the Region of Concepción (Chile). Two groups were formed: the experimental group, which followed a Chilean-adapted MedDiet supplemented with Chilean hazelnuts, and the control group, which adhered to a low-fat diet. The control group was selected as an active comparator following standard low-fat dietary recommendations for dyslipidaemia management according to Chilean Ministry of Health (MINSAL) guidelines. This pragmatic design was chosen for ethical reasons, as all participants required dietary treatment, and to compare two clinically relevant dietary strategies rather than isolate the effect of a single dietary component. The sample size was calculated based on changes in serum total cholesterol, using the parameters reported by Tey et al. studies [23,24], whose parallel-arm randomized trials, each lasting 84 days and including approximately 35 participants per group, used daily hazelnut doses ranging from 30 to 60 g. The calculation assumed the detection of a between-group difference of 0.32 mmol/L or greater in total serum cholesterol, with a standard deviation (SD) of 0.70 and a baseline-to-follow-up correlation of 0.80. Considering an anticipated dropout rate of 20%, approximately 50 participants per group (total n = 100) were estimated to be required. PBMC-derived oxidative stress and inflammatory markers were pre-specified as secondary mechanistic outcomes before the analyses were conducted; however, they were not included in the original sample size calculation. The study was therefore primarily powered for lipid outcomes, while PBMC analyses were intended to provide complementary mechanistic insight into redox and inflammatory modulation associated with the dietary intervention.

Block randomization was performed by an independent statistician to ensure sex balance across groups. Given the open-label nature of the dietary intervention, the nutritionists overseeing participant enrolment were aware of group assignments. The personnel performing biochemical analyses and the statistician conducting the analyses were blinded to group allocation to ensure unbiased assessment of outcomes. No patients or public representatives participated in the planning, implementation, or dissemination of this research.

Eligible participants were adults between 18 and 60 years of age presenting with elevated total blood cholesterol (TC > 200 mg/dL). Exclusion criteria included individuals under medical treatment for hypercholesterolemia, those with systemic diseases such as diabetes or hypertension, or previously diagnosed hepatic or renal disease, identified by a previous self-reported physician diagnosis and/or current use of disease-specific medication; pregnant or breastfeeding individuals, those with an immediate family member already enrolled in the study, individuals with nut allergies, and those with electronic implants, such as pacemakers. Prior to enrolment, all participants received a full explanation of the study procedures and gave their written informed consent voluntarily. The protocol adhered to the ethical principles of the Declaration of Helsinki and received approval from the Comité Ético Científico Servicio Salud Concepción (CEC-SSC: 21-09-48, Concepción, Chile). The trial was registered at ClinicalTrials.gov under the identifier NCT07087704 on 8 July 2025. No modifications were made to the original study protocol after the trial had begun.

For biochemical analysis, unless otherwise specified, all chemical reagents, activators, and solvents were purchased from Sigma-Aldrich Química Ltd. (Santiago, Chile).

### 2.2. Nutritional Intervention

Individual energy requirements were calculated using the FAO/WHO/UNU [25] predictive equation for resting metabolic rate, corrected for physical activity level. Both dietary plans were normocaloric, since the trial was not designed to induce weight loss, and were tailored to each participant’s food preferences, daily schedule, cultural background, and economic circumstances.

Participants in the control group adhered to a low-fat dietary pattern in which fat contributed less than 30% of total energy intake, protein approximately 15%, and carbohydrates between 45% and 55%, consistent with dyslipidaemia management guidelines issued by MINSAL [2]. The experimental group followed a Chilean-adapted Mediterranean dietary pattern based on Mediterranean principles but incorporating local Chilean foods, supplemented with 30 g/day of *G. avellana*. The total dietary pattern of the experimental group was designed to provide approximately 40% of energy from fat, 13% from protein, and 40–45% from carbohydrates. Both interventions complied with established limits for dietary cholesterol (<300 mg/day), sodium (<2.4 g/day), and added sugars (<10% of total energy intake).

The Chilean-adapted Mediterranean diet emphasized high consumption of fruits and vegetables (e.g., tomatoes, leafy greens, legumes, onions, and seasonal fruits), whole grains (e.g., whole wheat bread and oats), and legumes (e.g., lentils and beans), together with moderate intake of fish and white meat (poultry). The main added fat source was extra virgin olive oil, complemented by nuts and seeds, particularly Chilean hazelnuts. Intake of red and processed meats, refined sugars, and ultra-processed foods was limited. These dietary characteristics are consistent with the main features of the Mediterranean dietary pattern and its adaptation to the Chilean food context [26,27]. The experimental diet contained a higher content of MUFA and polyunsaturated fatty acids (PUFA), dietary fibre, micronutrients such as potassium and magnesium, as well as polyphenols derived from both plant-based foods and Chilean hazelnuts [20].

A qualified nutritionist assessed and quantified participants’ food intake through two 24 h dietary recalls conducted on non-consecutive days. Additionally, each participant attended three individual nutrition counselling sessions and participated in three group sessions throughout the intervention. Follow-up counselling was supported by printed materials (e.g., menus, portion guides, and practical tips). In the experimental group, 30 g/day of *G. avellana* was provided monthly in pre-portioned packages. All Chilean hazelnuts were purchased from the same local supplier, who sourced the nuts from different harvesters. Adherence was monitored through distribution logs and self-report. Both groups received identical counselling intensity and educational support, although no food products were provided to the control group. Possible adverse effects associated with the dietary interventions were monitored over the 6-month intervention period through both structured and informal evaluations conducted during the monthly counselling sessions. No serious adverse events were expected due to the non-pharmacological nature of the intervention, and safety was monitored throughout the study during follow-up visits.

### 2.3. PBMCs Purification

Blood samples were collected in vacutainer tubes containing EDTA (ethylenediaminetetraacetic acid) as an anticoagulant at baseline (day 0) and after six months of adherence to the assigned diet. Participants were instructed to fast for eight hours before sample collection, refrain from consuming liquids for two hours before sampling, and avoid intense physical activity for 48 h before blood collection. PBMC fractions were purified from whole blood following and adapting the method described by Boyum [28], using Ficoll-Paque Plus reagent (Cytiva, Santiago, Chile). Briefly, the blood–Ficoll gradient was centrifuged at 900× *g* (2200 rpm) for 30 min at room temperature with slow acceleration and deceleration. The PBMC fraction was collected, washed with PBS, and centrifuged again at 900× *g* (2200 rpm) for 10 min at 4 °C. PBMCs were divided into three aliquots: one sample was lysed with distilled water for enzymatic activities and malondialdehyde (MDA) levels, another sample was preserved with RIPA buffer for dot blot analysis (protein carbonyl and nitrotyrosine determination), and the other was used for RNA extraction. In addition, an aliquot was resuspended in PBS for reactive oxygen species (ROS) production. Lysed cells were stored at −80 °C until use. An aliquot of whole blood was analysed using an automatic flow cytometer system to determine lymphocyte, eosinophil, basophil, and monocyte counts to obtain the total number of PBMCs in each sample.

### 2.4. ROS Production

ROS production in PBMCs was evaluated using three activators: Zymosan A (ZA), lipopolysaccharide (LPS), and phorbol myristate acetate (PMA), with 2,7-dichlorofluorescein diacetate (DCFH-DA) as the indicator. Stock solutions were prepared as follows: DCFH-DA (30 µL/mL) in ethanol and Hank’s medium, ZA (1 mg/mL) in PBS, LPS (100 µg/mL) in PBS, and PMA (10 ng/mL) in DMSO. Each well of a black 96-well microplate received 50 µL of PBMC suspension, 50 µL of the activator, and DCFH-DA. Fluorescence (Ex: 485 nm; Em: 540 nm) was measured for one hour at 37 °C using a BioTek Synergy 2 Multi-Detection Microplate Reader (Biotek Instruments, Winooski, VT, USA) [29]. As DCFH-DA fluorescence is sensitive to a range of oxidant species and is not specific for H_2_O_2_, the results are referred to as ROS production rather than H_2_O_2_ production.

### 2.5. Malondialdehyde (MDA) Assay

MDA was quantified using a colorimetric assay. A standard curve of MDA was prepared, and samples were incubated with 1-methyl-2-phenylindole (10.3 mM) dissolved in acetonitrile:methanol (3:1) and HCl (12 N) at 45 °C for three hours. After centrifugation at 15,000× *g* for 10 min, the supernatant was transferred to a 96-well plate, and absorbance was read at 586 nm using a BioTek Synergy 2 Multi-Detection Microplate Reader (BioTek Instruments, Winooski, VT, USA) [29].

### 2.6. Protein Carbonyl and Nitrotyrosine Determination

Carbonyl and nitrotyrosine levels were measured using OxiSelect™ Protein Carbonyl and Nitrotyrosine Immunoblot Kits (Cell Biolabs, INC, LabTechnologies, Santiago, Chile) following the manufacturers’ protocols. Protein concentration was determined using the Pierce BCA Protein Assay Kit (ThermoFisher, Life Technologies, Santiago, Chile). Samples (10 µg for carbonyls and 150 µg for nitrotyrosine) were loaded onto PVDF membranes using the Dot Blot Hybridisation Manifold System (Life Technologies, Santiago, Chile). Chemiluminescence detection using luminol (SuperSignalTM West Pico PLUS Chemiluminescent Substrate, ThermoFisher, Life Technologies, Santiago, Chile) was performed using a C-DiGit^®^ Blot Scanner (LI-COR Biosciences, Lincoln, OR, USA). Images were analysed using Image Studio™ Lite software v6.0 (LI-COR Biosciences, Lincoln, OR, USA).

### 2.7. Enzyme Activities

The enzymatic activities of CAT, SOD, and glutathione reductase (GRd) in PBMCs were determined using a BioTek Synergy 2 Multi-Detection Microplate Reader (BioTek Instruments, Winooski, VT, USA) at 37 °C. CAT activity was assessed using a modified Aebi method (260 nm) [30], GRd activity was measured using a modified Goldberg method (339 nm) [31], and SOD activity was determined by an adaptation of the method described by Flohe (550 nm) [32].

### 2.8. PBMCs RNA Extraction and Real-Time qPCR Assay

Gene expression of *CAT*, *SOD*, *GRd*, interleukin-10 (*IL-10*), and tumour necrosis factor-alpha (*TNF-α*) was evaluated using RT-qPCR with a fluorescent reporter, using *β-Actin* as the reference gene. Total RNA was extracted from PBMCs using trizol (TRI Reagent^®^, Sigma-Aldrich Química Ltd., Santiago, Chile). RNA concentration was determined fluorometrically using a Qubit fluorometer, and RNA integrity was assessed by capillary electrophoresis using a Qsep1-Plus system (BiOptic Inc., New Taipei City, Taiwan). One microgram of total RNA was reverse-transcribed using the High-Capacity cDNA Reverse Transcription Kit in a final volume of 20 µL according to the manufacturer’s instructions. Reverse transcription (RT) and qPCR were conducted according to the manufacturer’s protocols, utilizing PowerTrack™ SYBR™ Green Master Mix (ThermoFisher, Life Technologies, Santiago, Chile) and a 7500 Fast Real-Time PCR System (Applied Biosystems, Waltham, MA, USA). The primers used for gene expression analysis were purchased from Fermelo Biotec (Santiago, Chile). Primers used and PCR conditions are listed in Table 1. All qPCR reactions were performed in technical triplicate. Amplification efficiencies were determined from standard curves generated using serial dilutions of cDNA and ranged from 90% to 105% for all evaluated assays, with comparable efficiencies between the target genes and β-Actin. Relative gene expression was calculated using the comparative Ct method (2^−ΔΔCt^), with *β-Actin* used for normalization and the corresponding baseline condition as the calibrator. *β-Actin* Ct values were examined across baseline and post-intervention samples and between dietary groups and showed no relevant variation under the experimental conditions, supporting its use as the reference gene [33]. RT-qPCR analyses were performed in 75 participants (43 in the experimental group and 32 in the control group), as some samples yielded insufficient RNA for successful amplification.

### 2.9. Statistical Analysis

Data were analysed using IBM SPSS Statistics v.29.0.1.0 (IBM Corp., Armonk, NY, USA) and expressed as mean ± standard deviation (SD). Normality was assessed using the Shapiro–Wilk test. Variables that did not follow a normal distribution were log-transformed prior to analysis. For ease of interpretation, untransformed data are presented in figures and tables. The effects of time, group, and the time × group interaction were evaluated using repeated-measures ANCOVA, with time as the within-subject factor and group as the between-subject factor, adjusted for age and sex. Pairwise comparisons were based on estimated marginal means with Bonferroni adjustment for multiple comparisons. Treatment effects were expressed as ratios of geometric mean changes (experimental vs. control) with 95% confidence intervals, obtained by back-transforming the group coefficient from linear regression models of log-transformed changes adjusted for age and sex. Ratios below 1 indicate a greater reduction (or smaller increase) in the experimental group. A two-sided *p*-value < 0.05 was considered statistically significant. Analyses were conducted on a complete-case basis, including only participants who completed the intervention. Missing outcome data were not imputed. A sensitivity analysis was performed using linear mixed models on log-transformed data, adjusted for age and sex, with an unstructured covariance matrix, including all randomized participants and all available observations under the missing-at-random assumption.

## 3. Results

A total of 235 patients were initially contacted for participation in the study. Of these, 129 were excluded for not meeting the inclusion criteria, including 114 based on TC measured in capillary blood and 15 based on TC measured in venous blood. Consequently, 106 subjects were enrolled and randomized in the study in Concepción, Chile. Enrolment occurred between February and March 2022, with the six-month follow-up period concluding in September 2022. No significant baseline differences were observed between the experimental and control groups in anthropometric variables, including body weight, body fat percentage, body mass index (BMI), and waist circumference [20]. During the study period, 25 participants discontinued the study. At the end of the intervention, the experimental group consisted of 47 participants, while the control group included 34 participants. In the experimental group, 5 participants were lost to follow-up because they stopped responding to telephone calls. In the control group, 11 participants stopped responding to telephone calls, 5 decided not to complete the study for personal reasons, and 1 was unable to complete the final assessment because of a prolonged trip abroad. No withdrawals were related to adverse effects or intolerance to the dietary interventions. A CONSORT flow diagram summarizing participant recruitment, allocation, follow-up, and analysis is provided in Figure 1.

The experimental group had an average age of 39.5 ± 12.2 years and consisted of 53.1% women and 46.8% men, while the control group had an average age of 38.8 ± 12.2 years, 67.6% women and 32.4% men. At baseline, participant characteristics indicated the presence of overweight in both groups (BMI: 28.2 ± 4.9 kg/m^2^ control group vs. 27.5 ± 4.8 kg/m^2^ experimental group, *p* = 0.514), hypercholesterolemia (241 ± 36 mg/dL control group vs. 247 ± 32 mg/dL experimental group, *p* = 0.439), normal glucose levels (86.8 ± 8.6 mg/dL control group vs. 86.0 ± 8.2 mg/dL experimental group, *p* = 0.690), and mildly elevated triglyceride levels (164 ± 67 mg/dL control group vs. 161 ± 74 mg/dL experimental group, *p* = 0.847). At baseline, dietary fat adequacy relative to the prescribed targets was 136.9 ± 6.3% in the control group and 123.5 ± 5.1% in the experimental group. No statistically significant differences were observed between groups at baseline, supporting comparability between the groups at the start of the study. The two groups followed an identical timetable of individual and group dietary counselling sessions, as described in the Section 2. No additional structured interventions were administered alongside the dietary protocols. Neither group reported adverse effects or unexpected clinical events during the six-month follow-up.

At the end of the intervention, caloric adequacy was 78.0 ± 3.5% in the control group and 98.0 ± 2.2% in the experimental group. Fat adequacy was 90.3 ± 4.9% and 114.5 ± 3.2%, protein adequacy was 78.3 ± 3.6% and 92.2 ± 2.7%, and carbohydrate adequacy was 76.2 ± 3.6% and 86.7 ± 2.1% in the control and experimental groups, respectively. Dietary intake data by major food groups are reported in detail in Ramos-Pardo et al. [20]. In the experimental group, all participants reported consuming the full daily portion of *G. avellana* provided throughout the intervention.

Figure 2A–C shows ROS production in PBMCs using different activators before and after the interventions. A significant time × group interaction was observed in PBMCs activated with LPS (ratio 0.76, 95% CI 0.59–0.97; T×G, *p* = 0.029). ROS production decreased significantly after the intervention in the experimental group, whereas the reduction observed in the control group did not reach statistical significance. For PBMCs stimulated with ZA, a significant effect of time (T, *p* < 0.001) was observed, with reduced ROS production after the intervention in both groups. ROS production decreased by 42.4% in the control group and by 55.2% in the experimental group, with a significant time × group interaction (ratio 0.68, 95% CI 0.52–0.89; T×G, *p* = 0.006). In contrast, in PBMCs stimulated with PMA, ROS production increased significantly in both groups after the intervention (50.0% in the control group and 29.6% in the experimental group). Although a significant overall time effect was observed (T, *p* < 0.001), no significant time × group interaction was detected (ratio 0.92, 95% CI 0.59–1.43; T×G, *p* = 0.708).

Oxidative damage biomarkers are presented in Figure 2D–F. PBMC MDA levels showed a significant reduction after the intervention in both groups (T, *p* < 0.001), decreasing by 55.2% in the control group and by 64.7% in the experimental group. The reduction was significantly greater in the experimental group, as indicated by a significant time × group interaction (ratio 0.62, 95% CI 0.43–0.89; T×G, *p* = 0.010). Protein carbonyl concentrations also decreased significantly after the intervention in both groups (T, *p* < 0.001). Carbonyl levels were reduced by 55.7% in the control group and by 58.2% in the experimental group, with no significant time × group interaction (ratio 1.04, 95% CI 0.56–1.92; T×G, *p* = 0.903). In contrast, nitrotyrosine levels decreased significantly in the control group (15.2%) (T, *p* = 0.042), whereas no significant changes were observed in the experimental group, with no significant time × group interaction (ratio 1.28, 95% CI 0.92–1.78; T×G, *p* = 0.141).

Antioxidant enzymatic activities are shown in Table 2. SOD activity increased significantly after the intervention in both groups (T, *p* < 0.001), with values rising by 72.7% in the control group and by 82.4% in the experimental group. However, no significant time × group interaction was observed (ratio 1.12, 95% CI 0.62–2.01; T×G, *p* = 0.702). Similarly, CAT activity increased markedly following the intervention in both groups (T, *p* < 0.001), increasing by 170% in the control group and by 142% in the experimental group. No significant time × group interaction was observed (ratio 0.77, 95% CI 0.56–1.06; T×G, *p* = 0.110). In contrast, GRd activity did not show significant changes over time, differences between groups, or a significant time × group interaction (ratio 1.04, 95% CI 0.76–1.42; T×G, *p* = 0.795).

At the expression level of these antioxidant enzymes (Table 2), statistically significant differences were found in both groups for the expression of *SOD* (T, *p* < 0.001), *CAT* (T, *p* < 0.001), and *GRd* (T, *p* < 0.001). *SOD* expression increased by 39.4% in the control group and by 28.2% in the experimental group, whereas *CAT* expression decreased by 38.6% and 42.3%, respectively. *GRd* expression increased by 49.9% in the control group and by 38.8% in the experimental group. No significant differences were observed between groups. The gene expression of *IL-10* and *TNF-α* showed no significant differences.

## 4. Discussion

According to clinical guidelines from MINSAL, the first action for managing hypercholesterolemia is a change in lifestyle habits, including a diet based on low-fat intake [2]. However, our findings suggest that a Chilean-adapted Mediterranean dietary pattern supplemented with Chilean hazelnuts produced substantial improvements in PBMC oxidative stress markers and antioxidant defences, with greater reductions in ZA- and LPS-stimulated ROS production and in MDA levels in the experimental group. These cellular effects are consistent with our previously reported findings from the same intervention, in which both dietary approaches improved the lipid profile, antioxidant status, and inflammatory biomarkers in plasma of hypercholesterolemic adults [20].

The comparable improvements observed between the two dietary interventions should also be interpreted in the context of the active comparator used in this trial. The low-fat diet is a structured dietary intervention based on clinical recommendations for dyslipidaemia management. Therefore, both groups were exposed to dietary counselling and modifications expected to improve overall diet quality. The absence of significant time × group interactions for most outcomes suggests that the addition of Chilean hazelnuts within a Chilean-adapted Mediterranean dietary pattern did not confer a measurable additional benefit over the low-fat dietary strategy for most PBMC redox and inflammatory markers, although these findings should be interpreted cautiously given that the study was primarily powered for lipid-related outcomes. However, these findings also suggest that improvements in redox-related markers can be achieved without necessarily restricting total dietary fat, provided that dietary fat quality and the overall dietary pattern are favourable. In this context, the cardiometabolic benefits observed with the incorporation of Chilean hazelnuts within a Chilean-adapted Mediterranean dietary pattern, and the associated contribution of unsaturated fatty acids and bioactive compounds from nuts and the overall dietary pattern, may represent a relevant alternative to fat-restricted dietary strategies. More broadly, achieving comparable outcomes through a dietary pattern that offers greater food variety and incorporates a native plant food with distinctive bioactive properties may provide a more palatable and culturally adapted approach to healthy eating, while encouraging greater consumption of whole plant foods and reducing less favourable dietary choices. Nevertheless, the greater reductions in ZA- and LPS-stimulated ROS production and in MDA levels in the experimental group may indicate a potential differential effect on specific oxidative pathways that warrants further investigation.

ROS production in PBMCs serves as a defensive mechanism against pathogens, regulated by various activation pathways [34,35]. However, excessive ROS production can induce oxidative damage to essential macromolecules such as lipids, proteins, and DNA [36,37]. In the context of cardiovascular diseases, oxidative stress contributes to the progression of atherosclerosis by promoting LDL oxidation and sustaining chronic inflammatory responses [38,39]. In the present study, we observed a significant reduction in ROS production in PBMCs activated with ZA in both groups, with a significant time × group interaction indicating a greater reduction in the experimental group. Similarly, PBMCs stimulated with LPS showed a significant time × group interaction, with a greater reduction in ROS production in the experimental group, suggesting that the Chilean-adapted Mediterranean dietary pattern enriched with Chilean hazelnuts may have exerted a differential effect on ROS generation under LPS stimulation. Although the underlying mechanism was not directly assessed in the present study, this effect could potentially involve modulation of Toll-like receptor (TLR)-4-dependent pathways. The phenolic compounds and oxylipins characterised in *G. avellana* have demonstrated antioxidant activity and inhibitory effects on pro-inflammatory enzymes [15], which may contribute to the attenuation of LPS-induced ROS generation observed in the experimental group. The activation of PBMCs mediated by ZA occurs through Dectin-1 and TLR2, triggering downstream signalling involving Syk and Src proteins [40,41,42,43]. On the other hand, PBMC activation through LPS occurs via the TLR4 receptor, which can promote NADPH oxidase activation and downstream ROS production [44,45,46]. Both pathways are closely linked to pro-inflammatory and oxidative responses, and TLR4-related signalling may therefore represent a potential pathway contributing to the differential response observed after LPS stimulation.

In contrast, PMA activates PBMCs through protein kinase C (PKC) by mimicking diacylglycerol, thereby stimulating cytokine production and mitochondrial ROS generation [47,48,49]. Interestingly, ROS production after PMA stimulation increased significantly in both groups, with no significant time × group interaction, suggesting that the response to this pharmacological stimulus was not modified by the type of diet. Overall, these results indicate that both dietary interventions maintained immune cell activation capacity while reducing ROS production in pathways more directly associated with inflammatory signalling.

Our study also reflects a decrease in the lipid oxidative damage marker, MDA, in both groups. This compound is a major byproduct of PUFA oxidation and can form adducts with proteins, thereby promoting further oxidative modifications [50]. Despite the experimental group having a higher intake of PUFAs, no increase in oxidative products was observed. On the contrary, the experimental group showed an even greater numerical reduction in MDA levels, suggesting that the higher unsaturated fat intake did not promote lipid oxidation under the conditions studied. These findings agree with the reductions observed in ROS production following ZA and LPS stimulation.

Similarly, protein carbonyl levels decreased significantly in both groups, supporting the notion of reduced oxidative modification of proteins. This finding is particularly relevant because lipid peroxidation products such as MDA can structurally modify ApoB-100 in LDL particles, contributing to the formation of oxidized LDL (ox-LDL) and the progression of atherosclerosis [51,52,53]. The simultaneous reduction in both MDA and protein carbonyls therefore reinforces the interpretation that oxidative damage was effectively attenuated by both dietary interventions. In contrast, nitrotyrosine concentrations decreased only in the control group, with no significant time x group interaction, suggesting that protein nitration pathways may be less sensitive to dietary modulation or may require longer intervention periods to exhibit consistent changes. Because nitrotyrosine formation may involve the interaction of nitric oxide and superoxide, direct assessment of these reactive species in future studies could help clarify the mechanisms underlying these findings.

In the present study, SOD and CAT enzymatic activities increased significantly in both groups, whereas GRd activity remained unchanged. Consistent with these findings at the plasma level, Ramos-Pardo et al. [20] reported a significant increase in plasma CAT activity in both dietary groups. At the transcriptional level, *SOD* and *GRd* expression increased after the intervention, while *CAT* expression decreased, indicating a partial dissociation between mRNA expression and enzymatic activity. This pattern suggests that antioxidant enzyme regulation may occur at multiple levels and that enhanced enzymatic activity is not necessarily dependent on increased gene transcription. Although not experimentally evaluated in the present study, post-transcriptional or post-translational regulatory mechanisms could potentially contribute to this discrepancy and may influence enzyme abundance or activity independently of changes in mRNA expression. Few studies have evaluated antioxidant enzymatic activity following nutritional interventions including hazelnuts; however, Monserrat-Mesquida et al. [54] described an increase in SOD and CAT activity at the end of their six-month MetDiet intervention with characteristics similar to our experimental design. Their subjects had CT > 200 mg/dL, meeting the hypercholesterolemia criteria defined in our study. In contrast, the increase in *GRd* expression was not accompanied by changes in its enzymatic activity, suggesting that the transcriptional response did not translate into greater catalytic capacity. This may indicate that basal GRd activity was already sufficient to maintain glutathione recycling under the studied conditions.

Regarding *IL-10* and *TNF-α* markers, no significant differences in gene expression were observed. The absence of changes in these inflammatory markers may indicate that the dietary interventions had a greater effect on antioxidant defences than on the expression of these specific inflammatory cytokines. This is in line with previous nutritional intervention studies showing that antioxidant-related biomarkers may respond more consistently than inflammatory cytokines, particularly in populations without marked baseline inflammation [55].

Overall, both dietary interventions were associated with favourable changes in redox-related markers in PBMCs. Both interventions reduced oxidative damage biomarkers and enhanced antioxidant enzyme activities, while changes in ROS generation varied according to the activation stimulus. Notably, the experimental diet produced a greater reduction in LPS-stimulated ROS production and in MDA levels, suggesting a potentially stronger modulatory effect on ROS generation under this specific inflammatory stimulus. These findings suggest that dietary patterns enriched in unsaturated fats and plant-based foods, including Chilean hazelnuts, may improve redox-related markers without detrimental effects on oxidative status. Our findings further support the concept that dietary quality, rather than fat restriction alone, may play a central role in the nutritional management of early-stage hypercholesterolemia. The absence of increased oxidative damage in the experimental group is particularly relevant given the higher intake of unsaturated fatty acids. This observation is consistent with previous evidence suggesting that nut consumption, especially hazelnuts, exerts beneficial effects on oxidative stress and cardiometabolic health. In this context, the meta-analysis by Brown, Ware and Tey [19] concluded that hazelnut consumption was associated with favourable antioxidant and lipid-related outcomes.

Among the strengths of this study is that it is the first, to our knowledge, to evaluate the effects of a Chilean-adapted Mediterranean dietary intervention supplemented with *G. avellana* on redox-related markers in PBMCs from hypercholesterolemic adults, highlighting the potential value of this native Chilean food within cardiometabolic nutrition strategies. However, several limitations should be acknowledged. The study was primarily powered for lipid-related outcomes rather than PBMC-derived mechanistic markers, which may have limited the ability to detect subtle between-group differences in oxidative and inflammatory parameters. Moreover, attrition differed between groups (37% in the control group vs. 10% in the intervention group), and the main repeated-measures analysis included only participants who completed the study (n = 81). However, a sensitivity analysis using linear mixed models including all randomized participants yielded consistent results, suggesting that differential attrition did not materially affect the conclusions. In addition, dietary adherence was partially assessed through self-reported dietary recalls, which may be subject to reporting bias. Finally, the chemical composition of the Chilean hazelnuts was not analysed during the intervention period, and natural variability in nutrient and bioactive compound composition cannot be excluded. Furthermore, a key limitation is that the experimental group received a comprehensive dietary intervention that differed from the control group in several components, including total fat content, fatty-acid composition, and consumption of fruits, vegetables, whole grains, legumes, olive oil, and 30 g/day of *G. avellana*. Therefore, it is not possible to determine whether the observed effects were attributable specifically to *G. avellana*, to the Chilean-adapted Mediterranean dietary pattern, or to their combination.

## 5. Conclusions

The high-fat Chilean-adapted MedDiet supplemented with Chilean hazelnuts produced beneficial effects on redox-related markers in PBMCs broadly comparable to those observed with the low-fat diet recommended by MINSAL clinical guidelines. Both dietary approaches reduced oxidative damage biomarkers and improved antioxidant defences, while the MedDiet enriched with Chilean hazelnuts was associated with a greater reduction in LPS-stimulated ROS production, suggesting a potential differential effect under specific inflammatory stimulation conditions. However, given the multifactorial nature of the experimental intervention, it is not possible to determine whether these effects are attributable specifically to Chilean hazelnut consumption, to the Chilean-adapted Mediterranean dietary pattern, or to their combination. These findings contribute to the growing body of evidence supporting the role of foods rich in healthy fats, including MUFAs and PUFAs, in favourably modulating oxidative status. Overall, our results support the feasibility of Chilean-adapted Mediterranean dietary patterns enriched with unsaturated fats and native plant foods as nutritional strategies for improving redox-related biomarkers in individuals with hypercholesterolemia, without requiring strict restriction of total dietary fat.

## Figures and Tables

**Figure 1:**
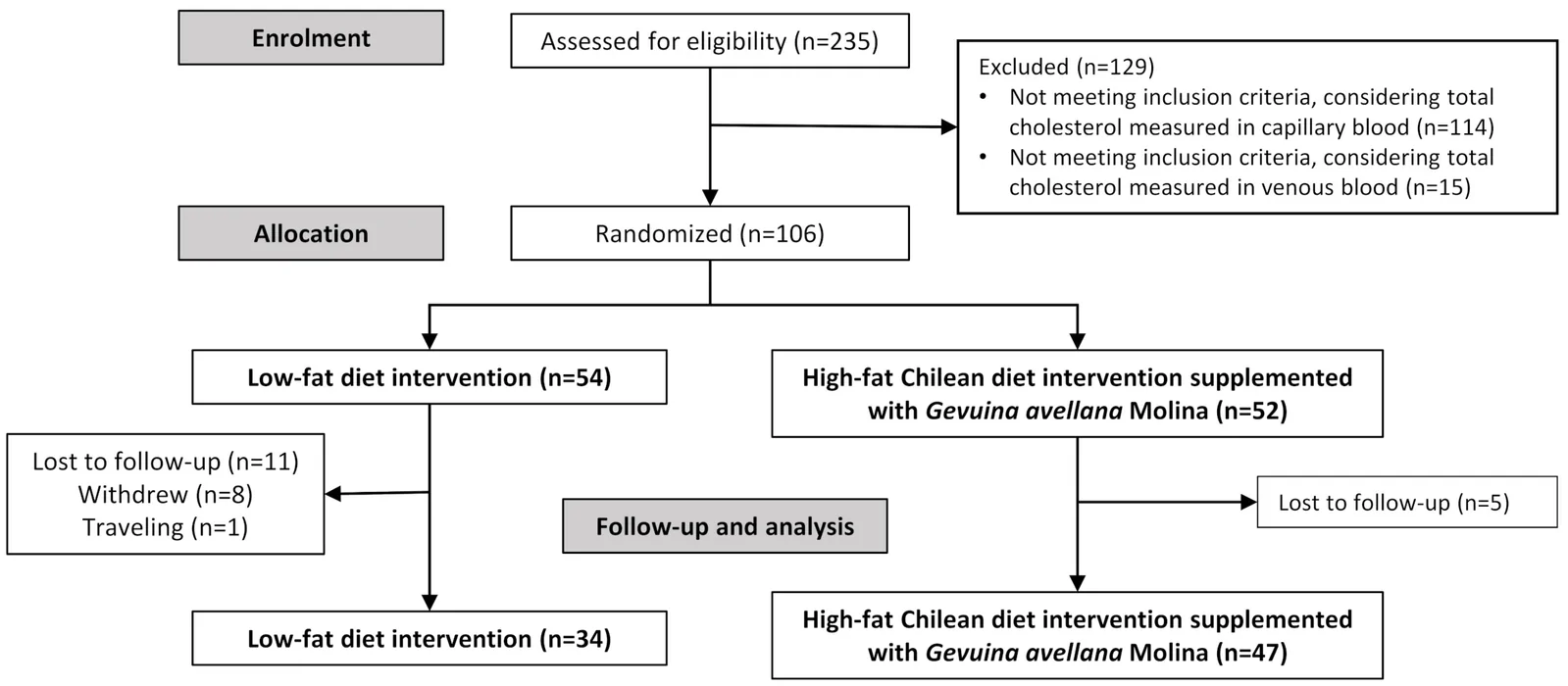
CONSORT flow diagram of participant recruitment and retention. Diagram showing the flow of participants from initial screening and eligibility assessment to enrollment, randomization, follow-up, and final analysis in the randomized controlled trial.

**Figure 2:**
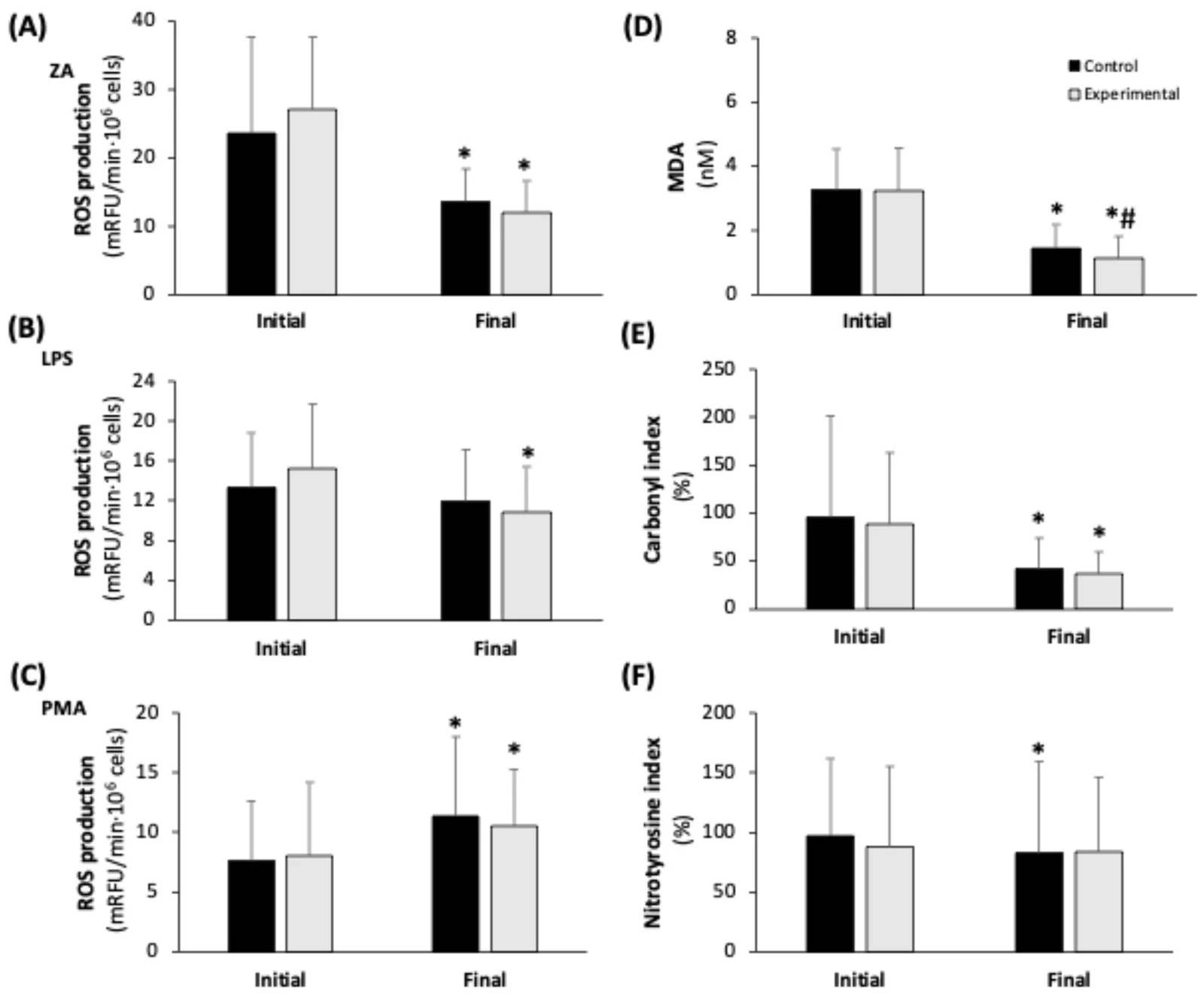
ROS production in PBMCs activated with (**A**) ZA (1 mg/mL), (**B**) LPS (100 µg/mL), and (**C**) PMA (10 ng/mL), and oxidative and nitrosative damage markers in PBMCs (**D**) MDA, (**E**) protein carbonyls, and (**F**) nitrotyrosine, before and after the intervention. Data are presented as mean ± SD, with n = 47 in the experimental group and n = 34 in the control group. Statistical comparisons were performed on log-transformed data using repeated-measures ANCOVA, with time as the within-subject factor and group as the between-subject factor, adjusted for age and sex. Pairwise comparisons were based on estimated marginal means with Bonferroni adjustment for multiple comparisons. * *p* < 0.05 compared with baseline within the same group. # *p* < 0.05 between the control and experimental groups at the same time point.

**Table 1:** Primers and conditions used in RT-qPCRs.

Gene	Primer	Conditions	
*CAT*	Fw: 5′-TTTGGCTACTTTGAGGTCAC-3′	95 °C	120 s
	Rv: 5′-TCCCCATTTGCATTAACCAG-3′	95 °C	5 s
		60 °C	30 s
*SOD*	Fw: 5′-TCAGGAGACCATTGCATCATT-3′	95 °C	120 s
	Rv: 5′-CGCTTTCCTGTCTTTGTACTTTCTTC-3′	95 °C	5 s
		60 °C	30 s
*GRd*	Fw: 5′-TTCCCGTGCAACCAGTTTG-3′	95 °C	120 s
	Rv: 5′-TTCACCTCGCACTTCTCGAA-3′	95 °C	5 s
		60 °C	30 s
*IL-10*	Fw: 5′-AGAACCTGAAGACCCTCAGGC-3′	95 °C	120 s
	Rv: 5′-CCACGGCCTTGCTCTTGTT-3′	95 °C	5 s
		60 °C	30 s
*TNF-α*	Fw: 5′-CCCAGGCAGTCAGATCATCTTCTCGGAA-3′	95 °C	120 s
	Rv: 5′-CTGGTTATCTCTCAGCTCCACGCCATT-3′	95 °C	5 s
		60 °C	30 s
*β-Actin*	Fw: 5′-ATTGCCGACAGGATGCAGAA-3′	95 °C	120 s
	Rv: 5′-GCTGATCCACATCTGCTGGAA-3′	95 °C	5 s
		60 °C	30 s

Abbreviations: CAT, catalase; SOD, superoxide dismutase; GRd, glutathione reductase; IL-10, interleukin-10; TNF-α, tumour necrosis factor-alpha; Fw, forward; Rv, reverse.

**Table 2:** Enzyme activities and gene expression in PBMCs before and after the intervention.

		Initial	Final	T	G	T×G
Enzyme activity						
SOD (pkat/10^9^ cells)	Control	0.066 ± 0.041	0.114 ± 0.067 *	<0.001	0.664	0.702
	Experimental	0.074 ± 0.052	0.135 ± 0.142 *			
CAT (K/10^9^ cells)	Control	157.1 ± 93.2	424.7 ± 226.5 *	<0.001	0.261	0.110
	Experimental	191.8 ± 84.2	464.7 ± 317.2 *			
GRd (mkat/10^9^ cells)	Control	69.5 ± 30.3	70.8 ± 37.8	0.441	0.592	0.795
	Experimental	71.3 ± 36.7	79.7 ± 46.0			
Gene expression						
SOD (%)	Control	100.0 ± 29.4	139.4 ± 62.6 *	0.677	0.029	0.760
	Experimental	92.2 ± 9.1	118.2 ± 28.7 *			
CAT (%)	Control	100.0 ± 43.7	61.4 ± 10.7 *	0.052	0.644	0.457
	Experimental	113.6 ± 48.1	65.5 ± 15.4 *			
GRd (%)	Control	100.0 ± 5.0	149.9 ± 58.7 *	0.329	0.432	0.335
	Experimental	101.5 ± 9.6	140.8 ± 55.5 *			
IL-10 (%)	Control	100.0 ± 14.6	107.7 ± 27.8	0.244	0.146	0.891
	Experimental	90.3 ± 10.4	99.3 ± 20.4			
TNF-α (%)	Control	100.0 ± 17.1	88.2 ± 13.7	0.742	0.630	0.860
	Experimental	96.5 ± 10.8	84.1 ± 7.8			

Data are presented as mean ± SD, with n = 47 in the experimental group and n = 34 in the control group for enzymatic activities, and n = 43 and n = 32, respectively, for gene expression analyses. Statistical comparisons were performed on log-transformed data using repeated-measures ANCOVA, with time as the within-subject factor and group as the between-subject factor, adjusted for age and sex. Pairwise comparisons were based on estimated marginal means with Bonferroni adjustment for multiple comparisons. * *p* < 0.05 compared with baseline within the same group. T, time effect; G, group effect; T×G, time × group interaction.

## Data Availability

There are restrictions on the availability of data for this trial, due to the signed consent agreements around data sharing, which only allow access to external researchers for studies following the project purposes. Researchers wishing to access the trial data used in this study can make a request to M.M.

## Abbreviations

The following abbreviations are used in this manuscript:

BMI	Body mass index
CAT	Catalase
CVDs	Cardiovascular diseases
DCFH-DA	2′,7′-Dichlorofluorescin diacetate
GRd	Glutathione reductase
H_2_O_2_	Hydrogen peroxide
HDL-C	High-density lipoprotein cholesterol
IL-10	Interleukin-10
LDL-C	Low-density lipoprotein cholesterol
LPS	Lipopolysaccharide
MDA	Malondialdehyde
MINSAL	Chilean Ministry of Health
MUFA	Monounsaturated fatty acids
PBMCs	Peripheral blood mononuclear cells
PMA	Phorbol 12-myristate 13-acetate
PUFA	Polyunsaturated fatty acids
ROS	Reactive oxygen species
RT-qPCR	Reverse transcription quantitative polymerase chain reaction
SFA	Saturated fatty acids
SOD	Superoxide dismutase
TC	Total cholesterol
TG	Triglycerides
TLR	Toll-like receptor
TNF-α	Tumor necrosis factor-alpha
ZA	Zymosan A

## References

[1] Mensah G.A., Fuster V., Murray C.J.L., Roth G.A., Global Burden of Cardiovascular Diseases, Risks Collaborators (2023). Global Burden of Cardiovascular Diseases and Risks, 1990–2022. J. Am. Coll. Cardiol..

[2] MINSAL (2018). Orientación Clínica Dislipidemias.

[3] MINSAL (2017). Encuesta Nacional de Salud (ENS) 2016–2017.

[4] Flaig B., Garza R., Singh B., Hamamah S., Covasa M. (2023). Treatment of Dyslipidemia through Targeted Therapy of Gut Microbiota. Nutrients.

[5] Pedro-Botet J., Climent E., Benaiges D. (2024). LDL cholesterol as a causal agent of atherosclerosis. Clin. Investig. Arterioscler..

[6] Mormone A., Tortorella G., Esposito F., Caturano A., Marrone A., Cozzolino D., Galiero R., Marfella R., Sasso F.C., Rinaldi L. (2024). Advances in Pharmacological Approaches for Managing Hypercholesterolemia: A Comprehensive Overview of Novel Treatments. Biomedicines.

[7] Chawla S., Tessarolo Silva F., Amaral Medeiros S., Mekary R.A., Radenkovic D. (2020). The Effect of Low-Fat and Low-Carbohydrate Diets on Weight Loss and Lipid Levels: A Systematic Review and Meta-Analysis. Nutrients.

[8] Raynor H., Wing R., Mela D.J. (2005). The effectiveness of popular diets: An overview. Food, Diet and Obesity.

[9] Daley S.F., Goldin J. (2026). Low-Fat Diet. StatPearls.

[10] Tosti V., Bertozzi B., Fontana L. (2018). Health Benefits of the Mediterranean Diet: Metabolic and Molecular Mechanisms. J. Gerontol. A Biol. Sci. Med. Sci..

[11] Martinez-Gonzalez M.A., Gea A., Ruiz-Canela M. (2019). The Mediterranean Diet and Cardiovascular Health. Circ. Res..

[12] Guasch-Ferre M., Willett W.C. (2021). The Mediterranean diet and health: A comprehensive overview. J. Intern Med..

[13] De Lira-Garcia C., Bacardi-Gascon M., Jimenez-Cruz A. (2012). Efectiveness of long-term consumption of nuts, seeds and seeds’ oil on glucose and lipid levels; systematic review. Nutr. Hosp..

[14] Arnesen E.K., Thorisdottir B., Bärebring L., Söderlund F., Nwaru B.I., Spielau U., Dierkes J., Ramel A., Lamberg-Allardt C., Åkesson A. (2023). Nuts and seeds consumption and risk of cardiovascular disease, type 2 diabetes and their risk factors: A systematic review and meta-analysis. Food Nutr. Res..

[15] Pino Ramos L.L., Jimenez-Aspee F., Theoduloz C., Burgos-Edwards A., Dominguez-Perles R., Oger C., Durand T., Gil-Izquierdo A., Bustamante L., Mardones C. (2019). Phenolic, oxylipin and fatty acid profiles of the Chilean hazelnut (*Gevuina avellana*): Antioxidant activity and inhibition of pro-inflammatory and metabolic syndrome-associated enzymes. Food Chem..

[16] Orem A., Yucesan F.B., Orem C., Akcan B., Kural B.V., Alasalvar C., Shahidi F. (2013). Hazelnut-enriched diet improves cardiovascular risk biomarkers beyond a lipid-lowering effect in hypercholesterolemic subjects. J. Clin. Lipidol..

[17] Deon V., Del Bo C., Guaraldi F., Abello F., Belviso S., Porrini M., Riso P., Guardamagna O. (2018). Effect of hazelnut on serum lipid profile and fatty acid composition of erythrocyte phospholipids in children and adolescents with primary hyperlipidemia: A randomized controlled trial. Clin. Nutr..

[18] Di Renzo L., Cioccoloni G., Bernardini S., Abenavoli L., Aiello V., Marchetti M., Cammarano A., Alipourfard I., Ceravolo I., Gratteri S. (2019). A Hazelnut-Enriched Diet Modulates Oxidative Stress and Inflammation Gene Expression without Weight Gain. Oxidative Med. Cell Longev..

[19] Brown R., Ware L., Tey S.L. (2022). Effects of Hazelnut Consumption on Cardiometabolic Risk Factors and Acceptance: A Systematic Review. Int. J. Environ. Res. Public Health.

[20] Ramos-Pardo S., Ramirez-Alarcon K., Gonzalez-Contreras C., Valdebenito E., Sureda A., Martorell M. (2026). Effect of a Chilean diet supplemented with *Gevuina avellana* Molina versus a low-fat diet on cardiometabolic, oxidative stress, and inflammatory biomarkers in Chilean adults with hypercholesterolemia: A randomized controlled trial. Nutr. Metab. Cardiovasc Dis..

[21] Costa A., Reynes B., Konieczna J., Martin M., Fiol M., Palou A., Romaguera D., Oliver P. (2021). Use of human PBMC to analyse the impact of obesity on lipid metabolism and metabolic status: A proof-of-concept pilot study. Sci. Rep..

[22] Sureda A., Martorell M., Bibiloni M.D.M., Bouzas C., Gallardo-Alfaro L., Mateos D., Capo X., Tur J.A., Pons A. (2020). Effect of Free Fatty Acids on Inflammatory Gene Expression and Hydrogen Peroxide Production by Ex Vivo Blood Mononuclear Cells. Nutrients.

[23] Tey S.L., Gray A.R., Chisholm A.W., Delahunty C.M., Brown R.C. (2013). The dose of hazelnuts influences acceptance and diet quality but not inflammatory markers and body composition in overweight and obese individuals. J. Nutr..

[24] Tey S.L., Brown R.C., Chisholm A.W., Delahunty C.M., Gray A.R., Williams S.M. (2011). Effects of different forms of hazelnuts on blood lipids and alpha-tocopherol concentrations in mildly hypercholesterolemic individuals. Eur. J. Clin. Nutr..

[25] FAO/WHO/UNU (2001). Human Energy Requirements.

[26] Echeverría G., Dussaillant C., McGee E.E., Mena C., Nitsche M.P., Urquiaga I., Bitran M., Pedrals N., Rigotti A. (2019). Promoting and Implementing the Mediterranean Diet in the Southern Hemisphere: The Chilean Experience. Eur. J. Clin. Nutr..

[27] Urquiaga I., Echeverría G., Dussaillant C., Rigotti A. (2017). [Origin, components and mechanisms of action of the Mediterranean diet]. Rev. Med. Chil..

[28] Boyum A. (1964). Separation of White Blood Cells. Nature.

[29] Capo X., Martorell M., Sureda A., Llompart I., Tur J.A., Pons A. (2015). Diet supplementation with DHA-enriched food in football players during training season enhances the mitochondrial antioxidant capabilities in blood mononuclear cells. Eur. J. Nutr..

[30] Aebi H. (1984). Catalase in vitro. Methods Enzymol..

[31] Goldberg D.M. (1984). Glutathione reductase. Methods Enzym. Anal..

[32] Flohe L., Otting F. (1984). Superoxide dismutase assays. Methods Enzymol..

[33] Roy J.G., McElhaney J.E., Verschoor C.P. (2020). Reliable reference genes for the quantification of mRNA in human T-cells and PBMCs stimulated with live influenza virus. BMC Immunol..

[34] Droge W. (2002). Free radicals in the physiological control of cell function. Physiol. Rev..

[35] Tan B.L., Norhaizan M.E., Liew W.P. (2018). Nutrients and Oxidative Stress: Friend or Foe?. Oxidative Med. Cell Longev..

[36] Salehi B., Martorell M., Arbiser J.L., Sureda A., Martins N., Maurya P.K., Sharifi-Rad M., Kumar P., Sharifi-Rad J. (2018). Antioxidants: Positive or Negative Actors?. Biomolecules.

[37] Rauf A., Khalil A.A., Awadallah S., Khan S.A., Abu-Izneid T., Kamran M., Hemeg H.A., Mubarak M.S., Khalid A., Wilairatana P. (2024). Reactive oxygen species in biological systems: Pathways, associated diseases, and potential inhibitors-A review. Food Sci. Nutr..

[38] Ito F., Sono Y., Ito T. (2019). Measurement and Clinical Significance of Lipid Peroxidation as a Biomarker of Oxidative Stress: Oxidative Stress in Diabetes, Atherosclerosis, and Chronic Inflammation. Antioxidants.

[39] Dubois-Deruy E., Peugnet V., Turkieh A., Pinet F. (2020). Oxidative Stress in Cardiovascular Diseases. Antioxidants.

[40] Baram L., Cohen-Kedar S., Spektor L., Elad H., Guzner-Gur H., Dotan I. (2014). Differential stimulation of peripheral blood mononuclear cells in Crohn’s disease by fungal glycans. J. Gastroenterol. Hepatol..

[41] Li J.T., Wang W.Q., Wang L., Liu N.N., Zhao Y.L., Zhu X.S., Liu Q.Q., Gao C.F., Yang A.G., Jia L.T. (2016). Subanesthetic isoflurane relieves zymosan-induced neutrophil inflammatory response by targeting NMDA glutamate receptor and Toll-like receptor 2 signaling. Oncotarget.

[42] Kozłowska E., Brzezińska-Błaszczyk E., Rasmus P., Żelechowska P. (2020). Fungal β-glucans and mannan stimulate peripheral blood mononuclear cells to cytokine production in Syk-dependent manner. Immunobiology.

[43] van Steenwijk H.P., Bast A., de Boer A. (2021). Immunomodulating Effects of Fungal Beta-Glucans: From Traditional Use to Medicine. Nutrients.

[44] Hua K.F., Chou J.C., Lam Y., Tasi Y.L., Chen A., Ka S.M., Fang Z., Liu M.L., Yang F.L., Yang Y.L. (2013). Polyenylpyrrole derivatives inhibit NLRP3 inflammasome activation and inflammatory mediator expression by reducing reactive oxygen species production and mitogen-activated protein kinase activation. PLoS ONE.

[45] Orecchioni M., Ghosheh Y., Pramod A.B., Ley K. (2019). Macrophage Polarization: Different Gene Signatures in M1(LPS+) vs. Classically and M2(LPS-) vs. Alternatively Activated Macrophages. Front. Immunol..

[46] Xu J., Núñez G. (2023). The NLRP3 inflammasome: Activation and regulation. Trends Biochem. Sci..

[47] Tran H.T.T., Herz C., Ruf P., Stetter R., Lamy E. (2018). Human T2R38 Bitter Taste Receptor Expression in Resting and Activated Lymphocytes. Front. Immunol..

[48] Mandal J.P., Shiue C.N., Chen Y.C., Lee M.C., Yang H.H., Chang H.H., Hu C.T., Liao P.C., Hui L.C., You R.I. (2021). PKCdelta mediates mitochondrial ROS generation and oxidation of HSP60 to relieve RKIP inhibition on MAPK pathway for HCC progression. Free Radic. Biol. Med..

[49] Tasli P.N. (2022). Usage of celery root exosome as an immune suppressant; Lipidomic characterization of apium graveolens originated exosomes and its suppressive effect on PMA/ionomycin mediated CD4(+) T lymphocyte activation. J. Food Biochem..

[50] Gegotek A., Skrzydlewska E. (2019). Biological effect of protein modifications by lipid peroxidation products. Chem. Phys. Lipids.

[51] Vandemoortele A., Babat P., Yakubu M., De Meulenaer B. (2020). Behavior of Malondialdehyde and Its Whey Protein Adducts during In Vitro Simulated Gastrointestinal Digestion. J. Agric. Food Chem..

[52] Videla L.A., Hernandez-Rodas M.C., Metherel A.H., Valenzuela R. (2022). Influence of the nutritional status and oxidative stress in the desaturation and elongation of n-3 and n-6 polyunsaturated fatty acids: Impact on non-alcoholic fatty liver disease. Prostaglandins Leukot. Essent. Fat. Acids.

[53] He Q., Chen Y., Wang Z., He H., Yu P. (2023). Cellular Uptake, Metabolism and Sensing of Long-Chain Fatty Acids. Front. Biosci. (Landmark Ed).

[54] Monserrat-Mesquida M., Quetglas-Llabres M., Bouzas C., Montemayor S., Mascaro C.M., Casares M., Llompart I., Ugarriza L., Martinez J.A., Tur J.A. (2022). Increased Adherence to the Mediterranean Diet after Lifestyle Intervention Improves Oxidative and Inflammatory Status in Patients with Non-Alcoholic Fatty Liver Disease. Antioxidants.

[55] Aleksandrova K., Koelman L., Rodrigues C.E. (2021). Dietary patterns and biomarkers of oxidative stress and inflammation: A systematic review of observational and intervention studies. Redox Biol..

